# Estimating copy number to determine *BRCA2* deletion status and to expect prognosis in localized prostate cancer

**DOI:** 10.1002/cam4.5617

**Published:** 2023-01-16

**Authors:** Takuhisa Nukaya, Makoto Sumitomo, Eiji Sugihara, Mayu Takeda, Sachio Nohara, Shigeki Tanishima, Masashi Takenaka, Kenji Zennami, Kiyoshi Takahara, Ryoichi Shiroki, Hideyuki Saya

**Affiliations:** ^1^ Fujita Cancer Center Fujita Health University Toyoake Japan; ^2^ Department of Urology School of Medicine Fujita Health University Toyoake Japan; ^3^ Department of Medical Research for Intractable Disease Fujita Health University Toyoake Japan; ^4^ Research Promotion Headquarters, Open Facility Center Fujita Health University Toyoake Japan; ^5^ Department of Bio Informatics, Communication Engineering Center, Electronic System Business Group Mitsubishi Electric Software Corp Tokyo Japan

**Keywords:** BRCA2, copy number, loss of heterozygosity, prognostic value, prostate cancer

## Abstract

**Background:**

The significance of *BRCA* alterations has been implicated in the development of metastatic castration‐resistant prostate cancer (PC). The details of the frequency and significance of *BRCA* alterations in localized PC remain unknown. In this study, we investigated the frequency and clinical significance of *BRCA* alterations in localized PCs using an in‐house next‐generation sequencer (NGS) system.

**Methods:**

DNA was extracted from formalin‐fixed paraffin‐embedded tissues of surgical specimens from 126 patients with clinically localized PC who underwent radical prostatectomy. The mutation information of 164 cancer genes was analyzed using the PleSSision‐Rapid test. Both copy number (CN) variation and loss of heterozygosity of various genes, such as *BRCA1* and *BRCA2*, were estimated and reported.

**Results:**

Next‐generation sequencer analyses revealed that the *BRCA2* CN was decreased in 17 patients (13.5%) and the *BRCA1* CN in six (4.8%) patients. NGS‐based CN values were shown to be highly correlated with droplet digital PCR‐based CN values. Tissue‐specific BRCA expression investigated using the Human Protein Atlas showed that the decreased CN of *BRCA2*, but not *BRCA1*, is responsible for the decreased BRCA activity in PC. Ten of the 22 patients with decreased *BRCA2* CN were presumed to have somatic heterozygous deletion. There were no observed associations between the heterozygous deletion of *BRCA2* and various clinicopathological parameters. Furthermore, three of 10 patients developed biochemical recurrence within 3 months after surgery. Multivariate analyses revealed that the initial prostate‐specific antigen levels and *BRCA2* CN were independent factors for biochemical recurrence.

**Conclusion:**

Our results suggest that a decrease in *BRCA2* CN may be used as a biomarker for predicting recurrence after surgery in localized PC. Early screening for somatic alterations in *BRCA2* using NGS may help to broadly predict the risk of PC progression.

## Graphical abstract

We showed a decrease in CN due to somatic BRCA2 deletion as a relatively frequent phenomenon that is linked to poor prognosis in PC. Early screening for BRCA2 somatic alterations using NGS may help to broadly predict the risk of PC progression, even if the patient does not carry a BRCA2 germline mutation. This will also provide opportunities for early intervention and targeted therapies.

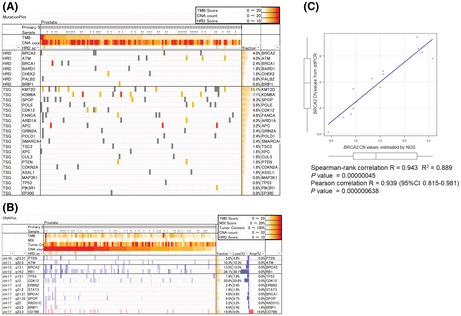

## 1 INTRODUCTION

Breast cancer 1 (*BRCA1*) and breast cancer 2 (*BRCA2*) genes play important roles in the homologous recombination repair (HRR) pathway by interacting with various other DNA repair proteins, such as ATM, CDK12, and CHEK2.^1^, ^2^ These pathways are frequently aberrant in cancer, which leads to accumulation of DNA damage and genomic instability. This phenomenon is called homologous recombination deficiency (HRD). Deletion of several *HRR* genes may increase the risk of various cancers, including prostate cancer (PC).^3^ Previous studies have suggested that more than 10% of patients with metastatic PC (mPC) have pathogenic *BRCA2* variants, half of which are derived from germline mutations.^4^, ^5^ A recent germline analysis study revealed that pathogenic *BRCA2* variants are significantly associated with risk of PC initiation and metastatic castration‐resistant PC (mCRPC).^6^, ^7^ Genomic aberrations in the HRR pathway, including those in *BRCA2*, are common in PC, particularly in advanced stages of mPC, and may be relevant for treatment stratification.^8^ Accordingly, poly ADP‐ribose polymerase (PARP) inhibitors have been reported to be a promising treatment strategy for patients with mCRPC who carry DNA repair mutations and are currently in clinical use.^9^

Hereditary PC with pathogenic *BRCA2* variants exhibits bi‐allelic BRCA2 inactivation by germline mutation and somatic loss of heterozygosity (LOH), while PC with somatic *BRCA2* alterations exhibits two‐hit alterations in the tumor. Several research groups have reported a higher frequency of biallelic deletion as a cause of somatic inactivation of BRCA2 in CRPC.^10^, ^11^ Moreover, recent studies suggest that there is no significant difference in the detection of HRR gene alterations, including *BRCA2*, when comparing samples from patients with mCRPC and treatment‐naive biopsies.^11^, ^12^ These findings suggest that the most observed alteration pattern of *BRCA2* in localized PC as well as CRPC is biallelic deletions. However, there remain several queries and limitations with respect to these findings. First, many research groups reported that heterozygous *BRCA2* deletion occurs more frequently compared to biallelic BRCA2 inactivation in PC.^12^, ^13^, ^14^ Second, high‐risk specimens that progress to CRPC were used in most studies reporting *BRCA2* alteration in localized PC, and there are a few studies reporting *BRCA2* alteration in surgically eligible PC specimens. Third, those studies did not consider the quantification of BRCA2 function, which may reflect HRD and PC development. Thus, the frequency and significance of BRCA2 inactivation in localized PC remain to be elucidated.

Next‐generation sequencing (NGS) has been broadly employed in clinical settings to identify multiple genomic alterations including single nucleotide variants and copy number (CN) variation (CNV). In the present study, we aimed to examine the frequency and clinical significance of HRR gene alterations, including *BRCA*, in localized PC using an in‐house NGS system. We have developed a method to quantify the degree of *BRCA2* deletion (or loss of function of BRCA2) in localized PC and assess its correlation with prognosis in clinically localized PC.

## 2 MATERIALS AND METHODS

### 2.1 Patient cohorts

The experimental protocols used in this study were approved by the Institutional Review Board of the Fujita Health University School of Medicine (approval numbers: HM21‐172). In addition, all methods were performed in accordance with the relevant local guidelines and regulations. Explanations to patients were also provided in writing. Moreover, a website with additional information that allowed for opt‐out was set up. A total of 126 patients with clinically localized PC who underwent robot‐assisted radical prostatectomy (RARP) between April 2021 and March 2022 at Fujita Health University Hospital were enrolled in this study.

### 2.2 Tissue‐specific BRCA protein expression

Tissue‐specific BRCA expression was investigated using The Human Protein Atlas (HPA, https://www.proteinatlas.org/).^15^, ^16^ HPA is a Sweden‐based program that integrates several omics technologies, including antibody‐based imaging, mass spectrometry‐based proteomics, transcriptomics, and systems biology, with the goal of mapping human proteins in cells, tissues, and organs. In the “tissue section,” the enrichment scores of tissue‐specific cell types of *BRCA1* and *BRCA2* genes were analyzed by predictive analysis of the expression specificity of a protein‐coding gene in a particular cell type by integrated co‐expression analysis. All data used in the analysis were obtained from RNA sequencing data of the Genotype‐Tissue Expression (GTEx) project portal (https://gtexportal.org/home/). For more details on this analysis and the classifications, see the “Tissue Cell Type” section of the HPA (https://www.proteinatlas.org/humanproteome/tissue+cell+type/method). The “pathology section” contains pathology‐related information based on mRNA and protein expression data of BRCA1 and BRCA2 from 17 different forms of human cancers, in addition to millions of in‐house generated immunohistochemically stained tissue section images. More information about the specific content, generation, and analysis of the data in the pathology section can be found in the methods summary data (https://www.proteinatlas.org/humanproteome/pathology/method).

### 2.3 NGS

Genomic testing was performed in‐house using the PleSSision‐Rapid‐Neo testing platform, with slight modifications to established methods (Fujita Health University Hospital, Toyoake, Japan).^17^, ^18^ The Maxwell RSC FFPE Plus DNA Kit (Cat. AS1720, Promega) was used to extract genomic DNA from 10‐μm‐thick formalin‐fixed paraffin‐embedded (FFPE) tissue sections of tumor or normal tissue specimens after RARP, according to the manufacturer's instructions. Genomic DNA was extracted, and the quality of DNA was verified by calculation of the DNA integrity number (DIN) using an Agilent 4200 TapeStation (Agilent Technologies); a threshold of DIN ≥2.0 was set for all analyses.

Sequencing libraries were then prepared by the targeted capture technique using the SureSelectXT Low Input Target Enrichment with Pre‐Capture Pooling (Agilent Technologies).^19^ Briefly, 10–100 ng of DNA extracted from FFPE tissues was enzymatically fragmented using the SureSelect Enzymatic Fragmentation Kit and SureSelect custom design panel (Agilent Technologies). Target regions of all 143 genes were specifically enriched using oligonucleotide probes. The overhung DNA fragments were then end‐repaired, adenylated, ligated to index/sequencing adapters, enriched by PCR, and purified according to the manufacturer's instructions. The quality and quantity of the purified pre‐capture library were evaluated using an Agilent 4200 TapeStation with D1000 ScreenTape (Agilent Technologies). Hybridization capture and library purification were performed according to the manufacturer's instructions. Briefly, 1.5 μg of pooled DNA from 16 pre‐capture libraries was used and 93.75 ng from each pre‐capture library was used to prepare the 16 library pools. The captured library pools were enriched by PCR and purified and quantified using an Agilent 4200 TapeStation with High Sensitivity D1000 ScreenTape (Agilent Technologies).

Enriched libraries were then sequenced by the paired‐end (150 bp × 2) sequencing method using the NextSeq 2000 NGS system (Illumina). Sequence data were analyzed as previously described using the GenomeJack bioinformatics pipeline (Mitsubishi Electric Software Corporation; http://genomejack.net/).^20^ Samples were rigorously inspected for quality, and those with tumor mean depth <200 and read remain rate <70% were subjected to reanalysis. The detailed method used to count CN is as follows.^21^ To calculate the baseline data used to correct the number of counts per amplicon, the number of reads arrayed per amplicon probe design region for each panel was counted and the reads per million (RPM) value (RPM arrayed reads) was calculated. The RPM coefficient of variation (CV), mean, and median of each amplicon were then tabulated for 48 FFPE samples from surgical specimens of renal cell carcinoma, for which competitive‐PCR had been performed previously in a preliminary study to quantify the CN of genes (including *BRCA2*). The median RPM for amplicons with CV < 0.32 and mean > 10 was then used as a baseline. The number of reads arrayed per panel amplicon probe design region was counted for each sample and the CN for each sample was calculated (calculation of CN values is necessary to calculate RPM values). Baseline ratios {log2 ratio [= log2 (sample RPM/baseline RPM. median)]}v in amplicons with CV < 0.32 and mean > 10 were tabulated. The overall standard deviation and median log2 ratio for each gene were then calculated: genes with a median log2 ratio greater than SD were amplification (amp)‐like, genes with a median log2 ratio > 2 SD were amp, genes with a median log2 ratio < −SD were loss‐like, genes with a median log2 ratio of those with a median log2 ratio < −2SD were classified as loss (heterozygous deletion or homozygous deletion). Finally, estimated CN values were adopted, which were recalculated to account for the effect of tumor content on the calculated CN values.

### 2.4 Droplet digital PCR

All droplet digital PCR (ddPCR) experiments were performed using QX200 ddPCR system in accordance with the manufacturer's instructions (Bio‐Rad).^22^ The following ddPCR primer kits were used: ddPCR™ Copy Number Assay: BRCA2, Human, *Homo sapiens* (Assay ID: dHsaCP250036) and ddPCR™ Copy Number Assay: RPP30, Human, *Homo sapiens* (Assay ID: dHsaCP2500350). Before analyzing the patient's DNA sample, a 381‐base pair genomic sequence encompassing *BRCA2* exon 13 (wild‐type and mutant alleles of unknown significance) and adjacent intron sequences were first cloned into a high‐copy bacterial plasmid to determine optimal thermal cycling conditions for allele‐specific binding of the fluorescent probe. Data analysis was performed using the accompanying platform software, QuantaSoftTM Analysis Pro (Bio‐Rad).

### 2.5 Statistical analyses

Statistical analyses were performed with EZR (Saitama Medical Center, Jichi Medical University), a graphical user interface for R (The R Foundation for Statistical Computing).^23^ Mann–Whitney and Fisher's exact tests were used for inter‐group comparisons. Correlations between parameters were evaluated using Spearman's rank correlation coefficient and Pearson's coefficient. Patients who presented two consecutive prostate‐specific antigen (PSA) levels of 0.2 ng/ml or higher after the postoperative PSA reached nadir were determined to have a biochemical recurrence (BCR). Kaplan–Meier analysis was used to examine the impact on biochemical progression‐free survival (bPFS), and statistical differences were ranked by the Mantel–Cox log‐rank test. Multivariate Cox proportional‐hazards models were used to examine variables associated with bPFS; *p*‐values < 0.05 were considered statistically significant.

## 3 RESULTS

### 3.1 Patient characteristics

Sequencing was performed for a total of 126 patients with clinically localized PC. Patient characteristics are shown in Table 1. Overall, the median age was 69 (range, 52–80) years, the median initial PSA level was 7.51 (range, 2.4–98.1) ng/ml, and the median Gleason score was 7 (range, 6–10). The clinical stages were T1c, 19; T2a, 62; T2b, 20; T2c, 13; T3a, 10; and T3b, 2. Median PFS was 183 days (range: 20–506) and median overall survival (OS) was 189 days (range: 20–506). Six (4.8%) tumors with BCR were identified during the observation period.

**TABLE 1:** Clinicopathological characteristics of 126 patients

	All (*n* = 126)
Age	
51–59	11
60–69	54
70–	61
Preoperative PSA (ng/ml)	
<10	85
10–20	33
>20	9
pT category	
pT2	99
pT3	26
pT4	1
Gleason grade sum	
≦3 + 3	3
3 + 4	51
4 + 3	43
≧4 + 4	29
pN category	
pN0	126
pN+	0
Resection margin status	
Negative	105
Positive	21

### 3.2 Pathogenic variant and CNV status of HRR genes in localized PC and reliability of NGS‐based *BRCA2* CN values

**FIGURE 1:**
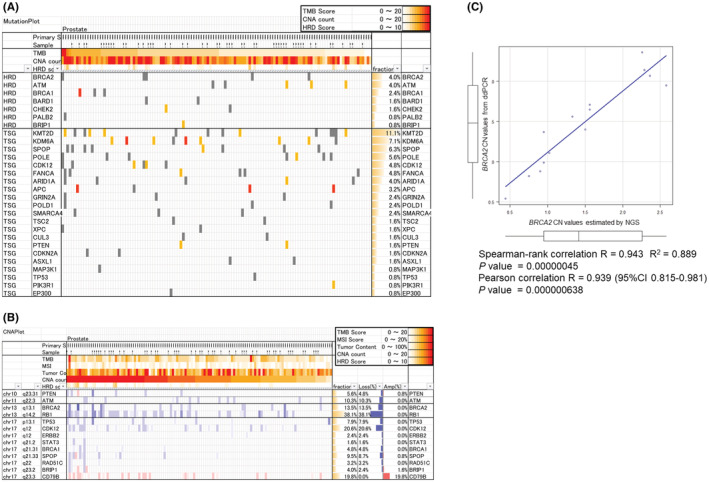
Mutation and CNV status of HRR and tumor suppressor genes from curation of NGS analyses and reliability of NGS‐based *BRCA2* CN values. (A) Among the genes responsible for HRD, the *ATM* pathogenic gene variant was identified in three cases, CDK12 in two and *BRCA1* in one. *BRCA2* variants were not identified. (B) CNV analysis showed that *CDK12* and *BRCA2* frequently had decreased CN (loss or loss‐like) in 19.7% and 12.9% of all cases, respectively, while *BRCA1* had a relatively low frequency of decreased CN (4.5% of all cases). (C) CN values of BRCA2 between NGS‐based CN data and droplet digital PCR (ddPCR)‐based CN data were compared. Statistical analyses using Spearman's rank correlation and Pearson's coefficients indicated that NGS‐based CN values correlated well with ddPCR‐based CN values.

Genomic DNA from 126 patients with PC undergoing RARP was successfully sequenced at an average sequencing depth of 768.5× (542×–1319×). As for the genes responsible for HRD, pathogenic gene variant in *ATM* was identified in three patients, *CDK12* in two patients and *BRCA1* in one patient. *BRCA2* variants were not identified (Figure 1A). The importance of pathogenic gene variants in tumor suppressor genes (TSGs), such as *TP53*, *RB1*, and *PTEN*, in PC carcinogenesis and progression has been previously suggested.^24^ However, in our study, pathogenic gene variants in *PTEN* were identified in two patients and *TP53* in one patient. *RB1* variants were not identified (Figure 1A).

Copy number variation analysis revealed decrease and increase in the CN of each gene, defined as loss and amplify, respectively. Our results showed frequent decrease in CN (loss or loss‐like) of *CDK12* in 26 (20.6%) patients and *BRCA2* in 17 (13.5%) patients, while the frequency of *BRCA1* CN decrease was relatively low as it was observed in only six patients (4.8%, Figure 1B). As for TSGs, reduction in the CN of *RB1*, *TP53*, and *PTEN* was observed in 48 (38.1%), 10 (7.9%), and 6 (4.8%) patients, respectively (Figure 1B, indicated in blue). *TP53* and *PTEN* were presumed to contain heterozygous or homozygous deletions in only two (1.6%) and four (3.2%) of the 126 patients, respectively (Figure 1B, indicated in dark blue).

To clarify the quantification of CN values measured by NGS, NGS‐based and ddPCR‐based CN values were compared. Fourteen of the 126 patients were selected for NGS‐based *BRCA2* CN evaluation: One patient had an NGS‐based value of <0.5, four patients had an NGS‐based value between 0.5 and 1.0, two patients had an NGS‐based value between 1.0 and 1.5, three patients had an NGS‐based value between 1.5 and 2.0, and four patients had an NGS‐based value of 2.0 or greater. Additionally, the *BRCA2* CN values of 14 patients were determined by ddPCR. Figure 1C shows the correlation of both datasets for the 14 cases. Statistical analyses using Spearman's rank correlation coefficient and Pearson's coefficient indicated that the NGS‐based CN values were highly correlated with the ddPCR‐based CN values (*p* = 0.00000045 and *p* = 0.000000638, respectively). Therefore, it is reasonable to employ NGS‐based *BRCA2* CN values as a means of determining the degree of *BRCA2* deletion.

### 3.3 BRCA function is predominantly dependent on BRCA2 in prostate tissues

**FIGURE 2:**
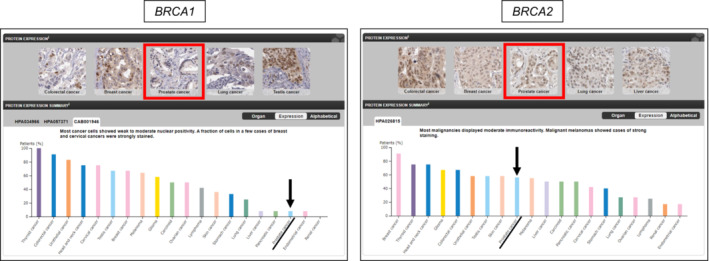
BRCA function is predominantly dependent on BRCA2 in PC tissues. Using the “pathology section” of HPA (https://www.proteinatlas.org), it was found that BRCA2 protein is expressed at higher levels in PC than BRCA1 protein. Arrows indicate the protein levels of BRCA1 (left) and BRCA2 (right) in PC.

**FIGURE 3:**
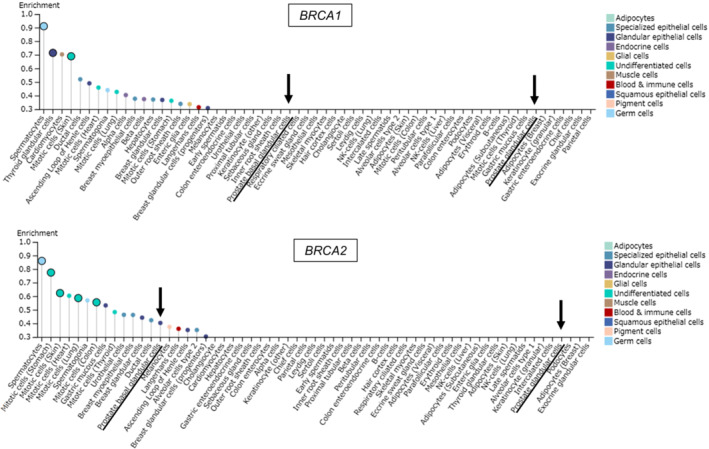
*BRCA2* RNA expression is dominant compared with *BRCA1* RNA expression in normal prostate tissues. Tissue‐specific *BRCA* RNA expression in prostate gland cells investigated using the “tissue section” of HPA. *Y*‐axis values show the enrichment score of expression of *BRCA1* and *BRCA2* in specific cell types found within the profiled tissues based on the tissue cell type section in the HPA (https://www.proteinatlas.org). Arrows indicate prostate basal glandular cells and prostate glandular cells, respectively.

Tissue‐specific BRCA expression was investigated using the HPA.^15^, ^16^ Using the “pathology section,” it was found that BRCA2 protein is expressed at higher levels in PC than BRCA1 protein (Figure 2). The results using “tissue section” predicted that *BRCA2* mRNA expression, but not *BRCA1* mRNA expression, was enriched in basal prostate gland cells (Figure 3). Our results suggest that BRCA function is predominantly dependent on BRCA2 in prostate tissues. Accordingly, we hypothesized that the decreased CN of *BRCA2*, but not *BRCA1*, is responsible for the decreased BRCA activity in PC.

### 3.4 Association between clinicopathological parameters and the presence or absence of *BRCA2* or *RB1* deletions on chromosome 13q, which are caused by somatic gene alterations

Previous studies have suggested a close association between the co‐deletion of *BRCA2* and *RB1*, which are in close proximity on chromosome 13q, and PC with poor prognosis and aggressive pathological features.^14^, ^25^ We therefore evaluated the frequency of *BRCA2* and *RB1* deletions and further examined the correlation of these deletions with pathological factors. CNV analyses confirmed that presumed deletions in *BRCA2* and *RB1* were present in 10 (7.9%) and 21 (16.7%) of the 126 patients, respectively (Table 2). There was no association between the presence or absence of *BRCA2* deletion and clinicopathological parameters, nor was there any association between the presence or absence of *RB1* deletion and clinicopathological parameters, which contradicted previous reports^14^, ^25^ (Table 2). Previous reports have shown that co‐deletion of BRCA2 and RB1 is a somatic heterozygous deletion.^14^, ^25^ We further examined the deletions by comparing the CN of both genes in normal tissues. NGS analyses revealed that the CN of *BRCA2* and *RB1* in adjacent normal prostate tissues was not decreased in 10 patients with PC with tumor *BRCA2* alteration (Table 3), suggesting that in the present study, *BRCA2* and/or *RB1* deletions were caused by somatic gene alterations. Co‐deletion of *BRCA2* and *RB1* was observed in eight of 10 patients. One patient was presumed to have a homozygous *BRCA2* deletion, while the others likely possessed heterozygous deletions. Of note, three of the 10 patients with *BRCA2* deletions were diagnosed with BCR within 3 months of RARP. One patient with BCR was presumed to not have a heterozygous *RB1* deletion (case 4; *RB1* CN in PC = 1.4, Table 3). Eleven patients with *RB1* deletions without *BRCA2* deletions were observed, but none had early BCR (data not shown). These results suggest that co‐deletion of *BRCA2* and *RB1*, most of which are attributed to somatic heterozygous deletions, is a frequent phenomenon in localized PC and that patients with *BRCA2* deletions frequently have poorer prognoses from localized PC. However, it is questionable whether *RB1* deletion itself is important in the prognosis of localized PC.

**TABLE 2:** Association between clinicopathological parameters and the presence or absence of *BRCA2* deletion or *RB1* deletion

	Without *BRCA2* deletion (*n* = 116)	With *BRCA2* deletion (*n* = 10)	*p* value	Without *RB1* deletion (*n* = 105)	With *RB1* deletion (*n* = 21)	*p* values
Median age (IQR)	69.0 (65–73)	67.5 (65.2–74)	0.863^a^	69.0 (65–73)	72 (66–74)	0.206^a^
Median iPSA, ng/ml (IQR)	7.56 (5.27–11.1)	5.84 (5.08–9.32)	0.414^a^	7.52 (5.3–11.1)	7.27 (4.86–10.0)	0.517^a^
pT						
pT2	93	6	0.277^b^	83	16	0.808^b^
pT3	22	4		21	5	
pT4	1	0		1	0	
Median Gleason sum (IQR)	7 (7–7)	7 (7–7)	0.394^a^	7 (7–7)	7 (7–8)	0.462^a^
pN						
pN0	116	10	1^b^	105	21	1^b^
pN+	0	0		0	0	
Surgical margin						
Negative	97	8	0.672^b^	88	17	0.752^b^
Positive	19	2				

^a^ Evaluated by Mann–Whitney test.

^b^ Evaluated by Fisher analysis.

**TABLE 3:** Association between somatic deletion of *BRCA2* and/or *PB1* and poor prognosis

Case	Age	iPSA	*BRCA2* CN in PC	*BRCA2* CN in NP	*RB1* CN In PC	*RB1* CN In NP	Co‐deletion	pT	GS	Outcomes
1	64	6.0	0.95; hetero	1.96	0.85	2.07	Yes	2c	4 + 4	
2	65	4.9	1.10; hetero	1.98	1.08	2.08	Yes	2c	3 + 4	
3	74	8.2	0.90; hetero	1.96	0.82	1.99	Yes	2c	4 + 3	BCR
4	65	5.7	0.44; homo	2.05	1.40	2.08	No	2c	4 + 3	BCR
5	66	20.4	1.02; hetero	1.98	0.92	1.99	Yes	3a	3 + 4	
6	74	4.0	0.75; hetero	1.91	0.86	1.91	Yes	2c	4 + 3	
7	66	22.3	1.33; hetero	2.00	1.95	2.20	No	3a	3 + 4	
8	74	9.7	1.12; hetero	2.20	1.03	2.13	Yes	2c	4 + 3	
9	68	5.4	0.95; hetero	2.07	0.96	2.11	Yes	3b	4 + 3	BCR
10	74	2.4	0.95; hetero	1.96	0.94	2.02	Yes	3a	4 + 3	

Abbreviations: hetero, heterozygous deletion; homo, homozygous deletion; NP, normal prostate tissue.

### 3.5 Associations between *BRCA2* deletion and patient prognosis

**FIGURE 4:**
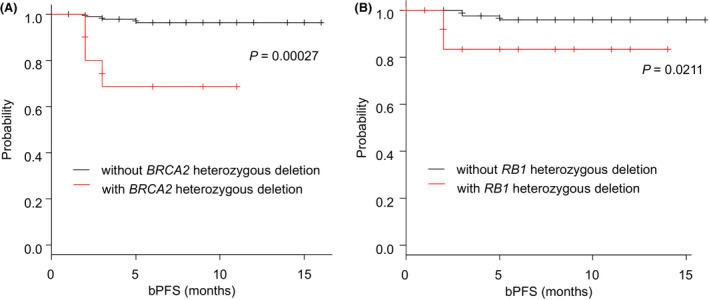
Associations between *BRCA2* deletion and patient prognosis. (A) Kaplan–Meier analysis of bPFS showed that group with *BRCA2* heterozygous deletion had poorer prognosis than the group without *BRCA2* heterozygous deletion (*p* < 0.001). (B) Kaplan–Meier analysis of bPFS showed that group with *RB1* heterozygous deletion had poorer prognosis than the group without *RB1* heterozygous deletion (*p* = 0.0211).

Since *BRCA2* and/or *RB1* deletion status has been suggested to be associated with poor prognosis in localized PC, we examined whether CN decrease could be a predictive marker of BCR in patients with localized PC. In this study, the median bPFS was 5.8 (95% confidence interval [CI], 5.1–6.5) months. Kaplan–Meier analysis of bPFS after surgery revealed that the group with *BRCA2* heterozygous deletion had significantly poorer prognosis (median PFS, 5.0 months; 95% CI, 2.7–7.2) than the group without *BRCA2* heterozygous deletion (median PFS, 5.9 months; 95% CI, 5.1–6.7; *p* < 0.001; Figure 4A). As noted in Table 3, it remains questionable whether *RB1* deletion itself is prognostically important in localized PC. Thus, the associations between *RB1* deletion and patient prognosis were also analyzed. Kaplan–Meier analysis showed that the group with *RB1* deletion had significantly poorer prognosis (median bPFS, 5.4 months; 95% CI, 3.5–7.4) than the group without *RB1* deletion (median bPFS, 5.9 months; 95% CI, 5.1–6.7; *p* < 0.05; Figure 4B). We then performed Cox proportional hazards model survival analysis using the CNs of *BRCA2*, *RB1*, *TP53*, and *PTEN* and various clinicopathological factors as candidate predictors of bPFS. The univariate analysis showed that the initial PSA (*p* = 0.006), extraprostatic extension (*p* = 0.049), *BRCA2* CN (*p* = 0.01), *RB1* CN (*p* = 0.034), and *TP53* CN (*p* = 0.041) were significantly associated with BCR, while the *PTEN* CN (*p* = 0.84) was not. In the multivariate analysis, the initial PSA (*p* = 0.001) and *BRCA2* CN (*p* = 0.033) remained associated with BCR, while the *RB1* CN (*p* = 0.622) and *TP53* CN (*p* = 0.167) did not (Table 4).

**TABLE 4:** Cox proportional hazard models for bPFS

Variable	Univariate		Multivariate	
	HR (95% CI)	*p*‐values	HR (95% CI)	*p*‐values
Age	0.959 (0.82–1.11)	0.582		
iPSA	1.049 (1.013–1.086)	0.006	1.07 (1.03–1.13)	0.001
GS	0.768 (0.195–3.024)	0.706		
pT3 over	4.158 (0.838–20.61)	0.081		
EPE positive (yes)	4.958 (1–24.57)	0.049	3.73 (0.64–21.42)	0.14
RM positive (yes)	2.499 (0.457–13.67)	0.29		
Tumor diameter	1.078 (0.98–1.18)	0.119		
*BRCA2* CN	0.12 (0.023–0.606)	0.01	0.09 (0.009–0.823)	0.033
*RB1* CN	0.23 (0.059–0.9)	0.034	0.56 (0.058–5.458)	0.622
*TP53* CN	0.07 (0.006–0.9)	0.041	0.12 (0.006–2.434)	0.167
*PTEN* CN	0.73 (0.035–15.29)	0.84		

Abbreviations: EPE, extraprostatic extension; RM, resected margin.

## 4 DISCUSSION

The results of our study revealed that heterozygous deletion due to a decrease in CN of *BRCA2*, estimated using our NGS system, is linked to early BCR in clinically localized PC. Notably, the *BRCA2* deletion status was not highly associated with the pathological parameters, such as Gleason scores and the pathological stage. Previous studies have suggested that co‐loss of *BRCA2* and *RB1*, located adjacent on chromosome 13q, is associated with poor PC prognosis.^14^, ^25^ Recently, Kluth et al.^14^ reported that 13q deletion is associated with high Gleason score, early BCR, and resistance to antiandrogen therapy. Chakraborty et al.^25^ reported that co‐loss of *BRCA2* and *RB1* induces epithelial–mesenchymal transition in human PC cell lines, LNCaP and LAPC4. Here, we showed that *RB1* and *BRCA2* deletions were associated with poor survival. Furthermore, reduction in the CN of *TP53* as well as *RB1* and *BRCA2*, which are suggested to be major factors in PC carcinogenesis and progression,^24^ was shown to be associated with poor survival, while only two (1.6%) of 126 patients with localized PC were suggested to have heterozygous or homozygous *TP53* deletions. The multivariate analyses revealed, however, that a decrease in *BRCA2* CN, but not in the CNs of *RB1* or *TP53*, was a strong predictor for early BCR after RARP. Our study highlights the significance and specificity of *BRCA2* CN, independent of other parameters, for the prediction of early BCR in patients with localized PC who underwent RARP.

In this study, we demonstrated that *BRCA2* heterozygous deletion is caused by somatic change without normal allele alterations in patients with localized PC. Theoretically, the biological behaviors between biallelic and heterozygous deletion of *BRCA2* in cancer progression should be different since BRCA2 function was not completely lost by the heterozygous deletion. However, Chakraborty et al.^25^ reported that heterozygous deletion of *BRCA2* significantly reduces BRCA2 protein levels in human PC cell lines, suggesting that the heterozygous deletion of *BRCA2* may be associated with a more aggressive phenotype of PC. This finding has recently received particular attention as “haploinsufficiency” in other cancer types.^26^ However, it is currently difficult to draw conclusions with respect to the differential prognosis between the heterozygous and the homozygous deletion of *BRCA2* in patients with PC. In patients with ovarian and breast cancer receiving platinum‐based adjuvant chemotherapy, patients lacking *BRCA* locus‐specific LOH had significantly worse OS than those with *BRCA2* homozygous deletion.^27^, ^28^ Although this result may seem contradictory from the perspective of loss of BRCA function, it is reasonable to assume that the effect of drugs acting on HRR increases with the absence of BRCA function. Accordingly, the loss of BRCA function may shorten disease‐free survival but increase drug sensitivity and improve OS. Thus, the biological differences between the homozygous and heterozygous deletions of *BRCA2* during PC progression need further investigation. Our preliminary results show that a patient with mCRPC, who lacked *BRCA2* locus‐specific LOH and had CN = 0.93, showed a poor PSA response to the PARP inhibitor olaparib, while another patient with mCRPC, who had homozygous *BRCA2* deletion and CN = 0.07, showed partial biochemical and radiological response to olaparib for more than 1 year. Notably, these two patients were both diagnosed with *BRCA2* loss in a comprehensive genomic profiling panel test (unpublished data). These results suggest that CN evaluation may be useful in precisely predicting the response to PARP inhibitors in mCRPC patients with *BRCA* alterations. Further testing in a larger cohort is needed to confirm the prognostic and therapeutic significance of *BRCA2* heterozygous loss in PC.

Similar to our results, several groups reported a significant fraction (around 10%) of patients with localized PC exhibited homozygous or heterozygous *BRCA2* deletion, which is equivalent to that in patients with mCRPC.^12^, ^14^, ^25^ This suggests that somatic *BRCA2* deletion occurs early in the tumorigenesis for patients with PC. Recent studies suggest that tumors with *BRCA2* deletions display pathological characteristics similar to those of intraductal carcinoma^29^ and neuroendocrine differentiated tumors,^30^ which are both poor prognostic indicators. Moreover, HRD with *BRCA2* deletions may lead to a pathogenic *MYC* variant, which is closely associated with the progression of localized or castration‐naïve PC to CRPC.^31^ Delayed diagnosis and treatment of patients with PC‐carrying *BRCA2* deletions can lead to the epithelial–mesenchymal transition, followed by rapid disease progression due to DNA damage and genomic instability.^26^ These findings strongly suggest that even heterozygous *BRCA2* deletions can cause HRD due to loss of function and may accelerate the progression of localized PC to BCR and eventually CRPC.

In the context mentioned above, it is crucial to establish an easy method to quantify the expression and function of BRCA2. In this study, we demonstrated that calculating the CN of *BRCA2* via NGS analysis is a reliable tool to reflect its loss of function. We believe that our demonstration of the reliability of BRCA2 CN calculations using our in‐house developed NGS platform is significant from the following perspectives. We showed that NGS, previously considered unsuitable for evaluating CN values, can be used to calculate CN with a reliability comparable to that of ddPCR. Furthermore, we demonstrated that our NGS approach can be used to analyze the variants and CNs of other important genes, such as *RB1* and *TP53*, at the same time as those of *BRCA2* in a rapid and cost‐effective manner, compared to other methods such as ddPCR. Our NGS approach may also be applicable to various other types of cancers for which other deletion scoring systems using fluorescence in situ hybridization and ddPCR have not been established. This approach may be particularly useful when limited amounts of biomaterial are available and screening for targeted genetic abnormalities for alternative therapies is necessary. Considering that the detection of heterozygous deletions using the FoundationOne CDx assay is only approved for ovarian cancer patients at the central laboratory,^32^ it is expected that the information obtained from our analysis will also contribute to expanding the therapeutic indications of this assay. We believe that *BRCA2* CN values determined by NGS can be added to practice guidelines regarding PC prognosis and HRR‐targeted therapy.

However, this study had a few limitations. First, this study reports the results of a single‐center study with a small number of cases and a short observation period. Second, we have not yet studied the expression and function of BRCA proteins in PC. Additional biological analysis will help in revealing the detailed relationship between the CNV and function of BRCA2. Further prospective multicenter studies will allow us to determine the clinical significance of the decrease in the CN of *BRCA2* in PC progression.

In conclusion, our findings identified a decrease in CN due to somatic *BRCA2* deletion is linked to poor prognosis in PC. Early screening for *BRCA2* somatic alterations using NGS may help to broadly predict the risk of PC progression, even if the patient does not carry a *BRCA2* germline mutation. This will also provide opportunities for early intervention and targeted therapies.

## AUTHOR CONTRIBUTIONS

**Takuhisa Nukaya:** Conceptualization (equal); formal analysis (equal); methodology (equal); visualization (equal); writing – original draft (equal); writing – review and editing (equal). **Makoto Sumitomo:** Conceptualization (lead); formal analysis (equal); investigation (equal); methodology (equal); project administration (equal); writing – original draft (lead). **Eiji Sugihara:** Data curation (equal); formal analysis (equal); methodology (equal); writing – review and editing (equal). **Mayu Takeda:** Data curation (equal); formal analysis (equal); methodology (equal); writing – review and editing (equal). **Sachio Nohara:** Data curation (equal); formal analysis (equal); investigation (equal); methodology (equal); writing – review and editing (equal). **Shigeki Tanishima:** Data curation (equal); formal analysis (equal); investigation (equal); methodology (equal); writing – review and editing (equal). **Masashi Takenaka:** Data curation (equal); investigation (equal); resources (equal); writing – review and editing (equal). **Kenji Zennami:** Data curation (equal); formal analysis (equal); investigation (equal); resources (equal); writing – review and editing (equal). **Kiyoshi Takahara:** Data curation (equal); formal analysis (equal); methodology (equal); resources (equal); writing – review and editing (equal). **Ryoichi Shiroki:** Conceptualization (equal); project administration (equal); resources (equal); supervision (equal); writing – review and editing (equal). **Hideyuki Saya:** Conceptualization (equal); methodology (equal); project administration (equal); supervision (equal); writing – original draft (equal); writing – review and editing (equal).

## CONFLICT OF INTEREST

Dr. Hideyuki Saya, the last author of this manuscript, is one of the associate editors of Cancer Science. No potential conflicts of interest were disclosed by other authors.

## ETHICAL APPROVAL

Ethical approval was obtained from the Institutional Review Board of the Fujita Health University School of Medicine prior to commencing this study.

## INFORMED CONSENT

Explanations to patients were also provided in writing. Moreover, a website with additional information that allowed for opt‐out was set up.

## Data Availability

None declared.
